# Increasing Performance of Professional Soccer Players and Elite Track and Field Athletes with Peak Performance Training and Biofeedback: A Pilot Study

**DOI:** 10.1007/s10484-016-9344-y

**Published:** 2016-10-19

**Authors:** Noortje H. Rijken, Remko Soer, Ewold de Maar, Hilco Prins, Wouter B. Teeuw, Jan Peuscher, Frits G. J. Oosterveld

**Affiliations:** 1 Expertise Group Ambient Intelligence, Saxion University of Applied Sciences, Enschede, The Netherlands; 2 Expertise Group Health and Wellbeing, Saxion University of Applied Sciences, M.H. Tromplaan 28, 7500 KB Enschede, The Netherlands; 3 Groningen Spine Center, University Medical Center Groningen, University of Groningen, Groningen, The Netherlands; 4 De Maar Coaching TCC, Glimmen, The Netherlands; 5 Heartmath Benelux, Meerssen, The Netherlands; 6 Research Group IT Innovations in Healthcare, Windesheim University of Applied Sciences, Zwolle, The Netherlands; 7 Twente Medical Systems International, Oldenzaal, The Netherlands

**Keywords:** Neurofeedback, Heart rate variability, Alpha power training, Sports performance, Soccer, Running, Mental stress

## Abstract

The aim of this pilot study was to investigate the effects of an intervention consisting of mental coaching combined with either electro encephalogram (EEG) alpha power feedback or heart rate variability (HRV) feedback on HRV, EEG outcomes and self-reported factors related to stress, performance, recovery and sleep quality in elite athletes. A prospective pilot study was performed with two distinct cohorts. Soccer players were provided with four sessions of mental coaching combined with daily HRV biofeedback (Group A); track and field athletes were provided with four sessions of mental coaching in combination with daily neurofeedback (Group B). Measurements were performed at baseline, post intervention and at 5 weeks follow-up. Objective measures: EEG and ECG. Subjective measures: Numeric Rating Scale for performance, Pittsburgh Sleep Quality Index, Rest and Stress Questionnaire and Sports Improvement-60. Group characteristics were too distinct to compare the interventions. Linear mixed models were used to analyze differences within groups over time. In Group A, significant changes over time were present in alpha power at 5 of 7 EEG locations (*p* < 0.01–0.03). LF/HF ratio significantly increased (*p* = 0.02) and the concentration (*p* = 0.02) and emotional scale (*p* = 0.03) of the SIM-60 increased significantly (*p* = 0.04). In Group B, the HRV low frequency power and recovery scale of the REST-Q significantly increased (*p* = 0.02 and <0.01 resp.). Other measures remained stable or improved non-significantly. A mental coaching program combined with either HRV or EEG alpha power feedback may increase HRV and alpha power and may lead to better performance-related outcomes and stress reduction. Further research is needed to elucidate the effects of either type of feedback and to compare effects with a control group.

## Introduction

Coping with mental stress and pressure to perform are clear demands for an optimal sports performance. Besides that, mental stress is considered to be an independent risk factor for sports injuries (American College of Sports Medicine et al. 2006; Junge 2000) and performance may be limited because of excessive mental stress, regardless of physical fitness. Therefore, there is an increasing interest in enhancement of psychological wellbeing for the improvement of sports performance (Hammermeister and VonGuenthner 2005). Several studies have suggested the effect of improved psychological wellbeing on sports performance (American College of Sports Medicine et al. 2006; Greenspan and Feltz 1989; Jones and Hardy 1989). In a review, 17 of 23 published studies reported positive performance effects after sports psychology interventions across many competitive sports (Greenspan and Feltz 1989). Most of these interventions emphasize the reduction of stress levels and sports injuries (Tranaeus et al. 2015). The reported effects however, are often small. A part of this may be explained by ceiling effects; athletes follow extensive training programs and their baseline fitness and health levels are high. Small effects may, however, be very worthwhile for athletes, since differences between competitors are very small and groups are very homogeneous (Paton and Hopkins 2005).

In the field of elite sports, different biofeedback systems are increasingly used to become aware of the (stress) physiology of the athletes’ bodies and to train athletes to influence stress responses and increase performance. A recent study has revealed small non-significant positive effects of alpha power neurofeedback training on performance in elite gymnasts, suggesting that this may be a worthwhile tool to use for performance enhancement (Dekker et al. 2014). Electroencephalography (EEG) can be used as a potential physiologic marker to evaluate stress. Particularly, the amount of alpha activity with EEG frequencies of 8–12 Hz is associated with cognitive performance and relaxation shown through decreased frontal EMG (Bazanova et al. 2013). Heart Rate Variability (HRV) is another psychophysiological indicator, which can be influenced by psychological interventions as well as by physical training. HRV indices are sensitive to performance effects of physical training in team sports players (Oliveira et al. 2013). Specifically, HRV biofeedback has been related to increases in HRV parameters and consequently, in performance in golf (Lagos et al. 2011), baseball (Strack 2003) and dance performance (Raymond et al. 2005). HRV-based biofeedback training has been found to decrease perceived stress and modify the activity of the autonomic nervous system in patients with cardiovascular diseases (McCraty et al. 2003; Moravec 2008; Nolan et al. 2005) and various chronic pain conditions (Tracy et al. 2016). HRV-based biofeedback is supposed to lead to increase in resilience, health (McCraty and Shaffer 2015), focus and recovery state.

According to theory, an increase in alpha power is suggested to be associated with improvement in cognitive performance, attention, and sleep, which can be useful in elite athletes (Cahn and Polich 2006; Kim et al. 2013) and there is evidence that alpha power can be increased after alpha neurofeedback training (van Boxtel et al. 2012). Furthermore, there are no studies that indicate which type of biofeedback (i.e. alpha power feedback or HRV feedback) works best, and who benefits most. The exact mechanisms on how changes in alpha power may be accomplished are largely unknown and the complex brain–heart connections, sympatico-vagal tuning as well as hormonal changes are suggested to contribute to these changes (Bazanova et al. 2014; Klimesch et al. 2007).

Hence, psychological interventions and biofeedback training may both have positive effects in training mental stress reactions and performance of elite athletes. Effects of a combined intervention of mental coaching with either HRV or EEG alpha power feedback have, to the awareness of the authors, never been described in literature, but it may be hypothesized that a combined intervention (mental coaching/biofeedback) leads to an increase of effect sizes and relevant decreases in mental stress and increase in performance.

The objective of this pilot study was to study the effects of an intervention consisting of mental coaching combined with either EEG alpha power feedback or HRV feedback on HRV or EEG outcomes and self-reported factors related to stress, performance, recovery and sleep quality in two distinct groups of elite sportsmen and women.

## Methods

### Participants

Two groups of participants were included in this study:

Group A: Professional soccer players from a soccer team performing at the highest soccer league in the Netherlands. There were no clear cut points for inclusion of participants. Therefore, indication for participation was made by the team’s medical staff. In general, indication was based on existence of stress limiting behavior and motivation of players. Participation in the study was then discussed with the players and was voluntary.

Group B: Elite athletes (sprinters and hurdlers) from different clubs performing at the highest Dutch competition levels. Subjects were recruited via the coaches. In general, indication was based on existence of stress limiting behavior and motivation of players. Participation was voluntary.

### Design and Procedure

**Fig. 1:**
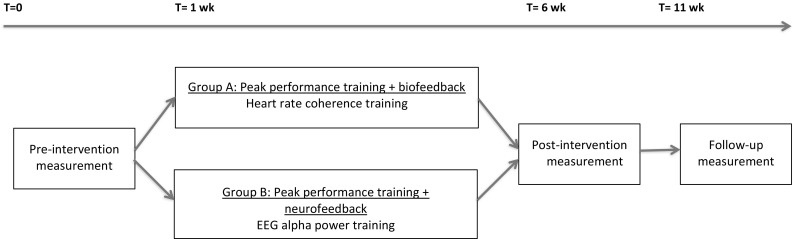
Flow chart of the study

An overview of the design can be found in Fig. 1. A pilot study was carried out with two cohorts. Both groups were trained separately with four sessions of mental coaching, which was combined with either heart rate variability (Group A) or alpha power (Group B) feedback. All subjects were instructed to train at a daily basis, at least 6 days a week. Training time and frequency were saved in a protected cloud. Athletes were not randomized and groups were not meant to compare. Subjects were measured at baseline, after the intervention and at 5 weeks follow up.

### Intervention

#### Mental Coaching Program

A mental coaching program was provided in the form of a peak performance program. The program consisted of four meetings, at week 1, 2, 3 and 5 in both groups. All meetings lasted 2.5 h. The peak performance program comprised a coaching program, which was based on the peak performance program of the HeartMath Institute (HeartMath 2004). The first sessions were focused on learning to become aware of stress and training of techniques with regards to breathing. In the second part of the training, participants were exposed to stress reactions, by focusing on individualized stress they experienced during their sports. This was done by several mental techniques and mindfulness-related exercises. Various mental techniques defined by the Heartmath Institute were applied such as the neutral technique, the quick coherence technique and the freeze frame technique (HeartMath 2013). The neutral technique is proposed as a mindfulness-related technique and forms the first step of the other techniques. It entails being attentive to the heart area and mentally imaging breathing in and out throughout the heart area. In the quick coherence technique, participants are encouraged to feel a positive emotion. In the freeze frame technique, an acute stressful event is recalled and subjects are being thought to recognize and stop the emotional response, and be able to choose a proper reaction. The training was provided by an experienced Heartmath licensed coach (EdM). Throughout the intervention period, participants were ought to train independently with biofeedback at home, at least 6 times a week.

#### Group A: HRV Feedback Training

Group A trained at home with the InnerBalance application developed by Heartmath, and home exercises were individually provided. Participants were instructed to train the mental techniques in a calm environment using HRV feedback. The application gave a breathing instruction and calculated heart coherence. A coherent heart rhythm is defined as a relatively harmonic, sine wave-like, signal with a very narrow, high-amplitude peak, in the low frequency (LF) region of the HRV power spectrum with no major peaks in the very low frequency (VLF) or upper portion of the high frequency (HF) regions. Coherence is defined by the maximum peak in the 0.04–0.26 Hz range of the HRV power spectrum, calculating the integral in a window 0.030 Hz wide, centered on the highest peak in that region, and then calculating the total power of the entire spectrum. The coherence ratio is formulated as: (Peak Power/[Total Power − Peak Power]). The coherence score is updated every 5 s (McCraty and Shaffer 2015). Coherence scores were presented by the InnerBalance application as a percentage of time in either high, medium or low coherence. A green box indicates the percent of time during which subjects are in a high coherence state, a blue box indicates the percent of time that patients are in a medium coherence state, and a red box indicates percent of time of incoherency, meaning increase stress. Summed, these boxes represent 100 % of the time. There is low quality evidence (uncontrolled pilot study) that training with the Heartmath techniques increases heart coherence (Soer et al. 2014, 2015). Because coherence was not a validated outcome measure before the appearance of the HRV-guideline, we choose not to use this as an outcome measure.

#### Group B: Alpha Power Feedback Training

The athletes trained at home with a neurofeedback system. Participants were provided with a tablet (Samsung galaxy Tab 10.1″) and a set of headphones (Philips, O’Neill stretch head band) that they used for listening to their favorite music. Participants all uploaded their own music on the tablet that was used for alpha power training. 5 Ag/AgCl ring electrodes (outer diameter: 12 mm; inner diameter: 5 mm) were mounted in the stretch headband and the ear covers of the headphone to measure EEG signals. The electrodes were positioned roughly over C3 measured against A1 and C4 measured against A2. A reference electrode was positioned over Cz. Tap water was used to optimize the contact between the electrodes and the skin. No impedance checks were made, but the quality of the data was assessed on-line via the ratio of the power in the 49**–**51 Hz (noise) range, and the power in the 4**–**35 Hz (signal) range. The signals were amplified (DC-400 Hz) and sampled at a rate of 1024 Hz by a 24 bit A/D converter on a MobiMini portable device (Twente Medical Systems international, Oldenzaal, The Netherlands). The signals were transmitted via Bluetooth to the tablets that controlled the experiment, and at the same time stored the data on a flash SD card for off-line analysis. The neurofeedback training used is based on a direct feedback mechanism on the alpha power in the participant’s individualized alpha frequency band (IAF ± 2 Hz), which is reflected by the quality of music that can be heard through a headset equipped with water-based electrodes. Low alpha power was reflected by “thin and distant” sound. The system has formerly been validated and is capable of significantly increasing the alpha power (van Boxtel et al. 2012). The applied training appeared to be “self-guided” (van Boxtel et al. 2012), which means that it did not involve any effort by the participants, but still resulted in an increase in alpha power.

### Measurements

Equal measurements were performed in both groups and existed of a quantitative EEG (qEEG) combined with ECG (qECG) session, which lasted 1.5 h. The measurement sessions were conducted in a dimly lit and quiet room that was not electrically shielded. Data were acquired using a TMSi 16 channel porti-system (Twente Medical Systems international, Oldenzaal, The Netherlands). qEEG was collected using a quickcap and 7 water electrode recordings from the following locations: C3, C4, P3, P4, O1, Oz and O2. EEG was recorded to the average reference in hardware and re-referenced in software to the algebraically linked ears. The sampling frequency was 2000 Hz. EEG signals were filtered in software using a high pass filter with a cut-off frequency of 0.9 Hz. Low pass filtering was 30 Hz. The ECG signals were recorded using a bipolar setup with two electrodes placed on the right and left flank and one ground-electrode placed on the left lower belly. ECG data was filtered using high pass filter of 1 Hz and a low pass filter of 200 Hz. The respiration was measured using an inductive belt and the respiration signal was filtered high pass (0.1 Hz) and low pass (5 Hz). The measurement configuration was equipped with two marker buttons for eyes open and eyes closed condition. Data were collected in polybench software (Twente Medical Systems International, Oldenzaal, The Netherlands). Because vision may influence alpha power, data were recorded during two periods of 5 min eyes open and eyes closed.

## Outcome Measures

Performance can considered a very multidimensional construct, depending on multiple factors. Therefore it was decided to administer both quantitative and subjective measures related to stress, recovery and sleep as well as heart and brain indices. Because there were no a priori hypothesis what could be the primary outcome measure, this study was considered a pilot with multiple outcome measures.

### Quantitative Analyses

#### EEG

Frequency analysis was performed on raw EEG signals using Fast Fourier Transformation (FFT). FFT power values were then transformed to a log_10_ scale and frequency components (0.25 Hz resolution) were averaged to define the alpha frequency band (IAF ± 2 Hz). IAF was determined for each individual by peak detection of the alpha frequency region. The frequency of the peak represents the IAF on the P3 Location, which was used for the individualized settings of the neurofeedback training application as well as for further analysis. Signal analysis in the alpha frequency domain during pre-training, post-training, and follow-up sessions was performed on data of the 5 min ‘eyes closed’ condition for standardization. Alpha power spectral density was estimated using the Welch (1967) method on digitally filtered EEG (Welch 1967). The EEG channels were filtered with a third-order Butterworth high-pass filter at 1 Hz and low-pass filter at 65 Hz. EEG spectra were computed and the EEG record was then segmented in 75 % overlapping intervals of 4 s. Thus, each epoch was shifted 1 s forward in time, and there were 300 segments in total. The segments were then transformed to the frequency domain using a Hanning window for tapering. The FFT power values were then transformed to a log_10_ scale and all frequency components (0.25 Hz resolution) were averaged to calculate the alpha frequency power. This value was calculated for all qEEG locations.

#### ECG

In the heart rate data, RR-intervals were recognized and analyzed in both the time and frequency domains. Regarding the time domain, SDNN was calculated as the standard deviation of the RR-intervals over the whole 5 min, expressed in seconds. RMSSD (s) represents the root of the mean squared successive differences between adjacent RR intervals over the entire recording. For the frequency domain measures, Fast Fourier transformation of the data was performed. The power of the peak in the low frequency region (0.04–0.15 Hz, with a central frequency around 0.1 Hz) and the high frequency region (0.15–0.4 Hz, with a central frequency at the respiratory rate around 0.25 Hz) were calculated. Low frequency power (LF), high frequency power (HF) and the ratio between LF and HF (LF/HF) of eyes closed tests were used as outcome measures for HRV (Malik and Chairman 1996).

### Subjective Outcome Measures

At all measurements, a comprehensive set of questionnaires was filled out to quantify sleep quality, recovery, stress and performance.

#### Sleep Quality

Sleep quality was assessed with the Pittsburgh Sleep Quality Index (PSQI). The PSQI is the most commonly used generic measure in clinical and research settings to measure the quality of sleep. The PSQI consists of 19 questions covering 7 domains (average Cronbachs α = 0.83): subjective sleep quality, sleep latency, sleep duration, habitual sleep efficiency, sleep disturbances, use of sleep medication, and daytime dysfunction over the last month. Lower scores reflect better sleep quality. Total score ranges from 0 to 21 with higher numbers indicating more sleep problems. A sum-score >5 indicates poor sleep (Buysse et al. 1989). Test–retest reliability of the PSQI is sufficient for group measurement, (ICCs > 0.70) and face and construct validity are sufficient in clinical and healthy persons (Mollayeva et al. 2015).

#### Recovery and Stress

The Recovery-Stress Questionnaire for athletes was used [RESTQ-Sport; (Kellmann and Kallus 2001)]. The RESTQ-Sport is a 76-item self-report measurement tool indicating general stress and recovery along with sport-specific stress and recovery. Score range per item of the RESTQ is from 0 (never) to 6 (always). Higher scores mean better recovery and less stress. The RESTQ-Sport identifies current stress/recovery states of athletes. Internal consistency is good, (Cronbach’s α values range from 0.72 to 0.93). Test–retest reliability after three days is sufficient with ICC > 0.7 for most scales (Kellmann and Kallus 2001).

#### Sports Improvement Measurement-60

The Sports Improvement Measurement-60 (SIM-60) was used to monitor sports improvement and has been designed for athletes. Different constructs can be identified: physical wellbeing, concentration capacity, coping, emotional stability, intuition and creativity, social wellbeing, performance and goal-oriented. For the current study, physical wellbeing, concentration capacity, coping and emotional stability have been used for analysis (Schüsler-van Hees and Schüsler 2011). Psychometric properties of the SIM-60 are currently being evaluated.

#### Performance

Self-reported performance was self-scored using a numeric 0–10 rating scale (NRS score) with 0 indicating worst performance and 10 indicating best performance. Numeric rating scales are available in multiple forms for differing purposes. In general, the NRS is a reliable, valid and responsive measure (Hjermstad et al. 2011).

### Statistics

In this exploratory study, missing values were not imputed. Data of both groups were analyzed separately. To determine whether outcomes changed during and after the training, linear mixed model analyses were performed, in which the dependent variables were the subjective self-reports and objective EEG and HRV measures. Because this was considered a pilot study, fixed slopes and intercepts were used. Cohen’s d values were calculated to indicate effect sizes during the intervention period (T0–T1) to interpret the relevance of the changes. Cohen’s d effect sizes were interpreted as follows: d < 0.5: small effect, d between 0.5 and 0.8: medium effect and d > 0.8: large effect (Cohen 1988). Additionally, the percentage of athletes who increased on the corresponding outcome during the intervention period were provided. Statistics were calculated with SPSS, version 22. A *p* value <0.05 was considered to be significant.

## Results

Group A consisted of 11 subjects and Group B of 10. In group B, one subject was lost at T2 and of two subjects one EEG measurement was missing because of insufficient signal quality because of woolly haired persons. Of the remaining participants, data were complete. Baseline characteristics of both cohorts are described in Table 1.

**Table 1:** Subject characteristics

	Group	
	Soccer (n = 11)	Athletes (n = 10)
Age [median (range)]	22 (21–32)	18 (16–38)
Gender (m/f)	11/0	8/2

### Group A

The aim was to practice 6 days per week, 3 times per day during the intervention period. Players records of practice were saved in a mean of 94 sessions per player (range 0–189 sessions). One session took for about 3 min. A number of players stated that not all practices were saved or they practiced without the Emwave software. The total Mean outcomes of Group A are listed in Table 2. Group A increased significantly in the SIM-60 emotional stability and concentration subscales, HRV LF/HF ratio and EEG alpha power on C3, C4, OZ, P3 and P4. Of 20 hypothesis tested, 8 improved significantly and 17 of 20 hypotheses had positive effect sizes varying between 0.00 and 1.13. On an individual level, soccer players improved between 40 and 100 % on outcomes. The increase of the LF/HF ratio appeared to be both because of an increase in LF power of 0.74 at T0–1.51 at T1, and a decrease in HF power from 0.53 at T0–0.23 at T1. This indicated a shift towards baroreceptor activity (McCraty and Shaffer 2015).

**Table 2:** Outcomes and linear mixed model analyses of soccer players (Group A)

Outcome	T0 [mean (SD)]	T1 [mean (SD)]	T2 [mean (SD)]	F (*df*)	*P*	Cohen’s d^a^	% improvement
Self report							
NRS	6.5 (0.42)	7.0 (0.88)	6.6 (1.17)	2.1(2,19)	0.15	0.84	64
PSQI	3.6 (2.42)	3.4 (1.36)	3.4 (2.17)	0.2 (2,19)	0.82	0.14	40
SIM-60 physical	69.6 (11.50)	70.4 (11.93)	69.0 (14.69)	0.1 (2,19)	0.94	0.08	55
SIM-60 coping	59.0 (9.65)	59.0 (9.69)	63.3 (10.56)	1.8 (2,19)	0.20	0.00	45
SIM-60 emotional	65.1 (10.50)	67.6 (8.41)	71.0 (8.49)	4.4 (2,19)	0.03	0.26	55
SIM-60 concentration	60.6 (10.09)	67.3 (9.25)	65.6 (11.90)	4.9 (2,19)	0.02	0.69	64
REST-Q stress	154.4 (13.94)	156.0 (11.25)	158.8 (27.78)	0.4 (2,19)	0.65	0.13	64
REST-Q recovery	97.9 (7.57)	102.8 (6.9)	101.9 (18.71)	0.5 (2,19)	0.59	0.68	73
Heart rate variability							
SDNN (s)	0.070 (0.034)	0.077 (0.034)	0.080 (0.028)	0.26 (2,20)	0.77	0.22	73
RMSSD (s)	0.055 (0.042)	0.055 (0.032)	0.055 (0.024)	0.00 (2,20)	1.00	0.00	64
Low frequency power	0.74 (1.07)	1.51 (1.22)	1.49 (0.98)	1.62 (2,20)	0.22	0.70	64
High frequency power	0.53 (0.89)	0.23 (0.19)	0.23 (0.19)	1.02 (2,20)	0.38	0.49	55
LF/HF ratio	2.53 (2.91)	7.52 (5.90)	9.81 (7.71)	4.84(2,20)	0.02	1.13	91
Alpha power							
Power C3	−1.99 (0.22)	−1.87 (0.17)	−1.82 (0.33)	4.41 (2,19)	0.03	0.61	90
Power C4	−2.02 (0.22)	−1.85 (0.18)	−1.79 (0.29)	7.91 (2,19)	<0.01	0.85	100
Power O1	−1.98 (0.39)	−1.79 (0.37)	−1.77 (0.45)	1.77 (2,19)	0.20	0.50	73
Power O2	−2.01 (0.37)	−2.01 (0.34)	−1.88 (0.47)	0.69 (2,17)	0.51	0.00	60
Power OZ	−2.23 (0.23)	−2.05 (0.32)	−2.02 (0.42)	4.21 (2,19)	0.03	0.65	91
Power P3	−2.26 (0.21)	−2.09 (0.30)	−2.04 (0.44)	4.45 (2,19)	0.03	0.66	73
Power P4	−2.25 (0.21)	−1.97 (0.33)	−2.08 (0.32)	5.18 (2,19)	0.02	1.01	91

^a^Cohens d calculated between T0 and T1. Positive Cohen’s d mean improved function; %improvement: % of athletes who increased between T0 and T1

### Follow-Up

At the five-week follow-up (T2), no significant declines in subjective and objective outcome parameters were detected compared to T1, indicating that effects lasted after the intervention period for at least five weeks. The frequency domain LF and HF and LF/HF ratio remained stable, and a further (slight) increase in Alpha power was observed at T2 compared to T1.

### Group B

Compliance: The aim for each participant was to practice 20 times at home during the intervention period. One practice session took for 30 min. A mean of 14.8 times were actually practiced, in which 6 participants practiced at least 17 times. Two participants had technical problems and two participants had compliance problems. Group B improved significantly on the recovery scale of the RESTQ and on HRV low frequency power. Results are presented in Table 3. Other measures did not improve significantly but most showed trends towards improvement. Of 20 hypotheses tested, 15 had positive effect sizes varying between −0.15 and 1.12. On an individual level, athletes improved between 40 and 89 % on outcomes. The LF power significantly increased, whereas the HF power did not, indicating that a change occurred in baroreceptor activity.

**Table 3:** Outcomes and linear mixed model analyses of athletes (Group B)

Outcome	T1 [mean (SD)]	T2 [mean (SD)]	T1 [mean (SD)]	F(*df*)	*P*	Cohen’s d^a^	% improvement
Self report							
NRS	6.4 (0.99)	7.2 (1.48)	6.3 (1.74)	1.97 (2,16)	0.17	0.64	89
PSQI	5.1 (3.24)	3.6 (2.07)	4.1 (2.28)	2.23 (2,18)	0.13	0.55	50
SIM-60 physical	64.0 (13.9)	63.0 (13.6)	65.5 (15.0)	0.12 (2,18)	0.89	-0.07	40
SIM-60 coping	61.7 (11.8)	66.8 (6.8)	65.9 (10.8)	2.19 (2,18)	0.14	0.53	50
SIM-60 emotional	69.1 (16.2)	70.9 (13.5)	70.7 (13.7)	0.32 (2,18)	0.73	0.12	70
SIM-60 concentration	65.1 (11.2)	65.4 (10.3)	67.1 (11.5)	0.23 (2,18)	0.79	0.03	60
REST-Q stress	151.8(18.1)	154.1 (19.6)	152.9 (23.0)	0.06 (2,18)	0.94	0.12	50
REST-Q recovery	92.2 (11.6)	99.4 (5.0)	99.6 (10.5)	5.47 (2,18)	0.01	0.81	80
Heart rate variability							
SDNN (s)	0.081 (0.056)	0.099 (0.048)	0.086 (0.035)	0.40 (2,18)	0.68	0.36	80
RMSSD (s)	0.079 (0.063)	0.091 (0.063)	0.075 (0.051)	0.18 (2,18)	0.84	0.20	60
Low frequency power	0.50 (0.59)	1.80 (1.63)	0.82 (0.66)	3.85 (2,18)	0.04	1.12	70
High frequency power	0.92 (1.05)	1.10 (1.44)	0.94 (1.19)	0.06 (2,18)	0.94	-0.15	40
LF/HF ratio	0.81 (0.72)	5.90 (8.81)	5.70 (8.25)	1.50 (2,18)	0.25	0.86	60
Alpha power							
Power C3	−1.70 (0.23)	−1.71 (0.34)	−1.66 (0.31)	0.25 (2,15)	0.79	−0.04	56
Power C4	−1.70 (0.26)	−1.67 (0.32)	−1.70 (0.32)	0.53 (2,15)	0.60	0.09	67
Power O1	−1.62 (0.55)	−1.56 (0.49)	−1.54 (0.55)	0.74 (2,15)	0.49	−0.10	75
Power O2	−1.67 (0.48)	−1.58 (0.54)	−1.49 (0.42)	1.29 (2,16)	0.30	−0.10	78
Power OZ	−1.90 (0.33)	−1.68 (0.39)	−1.82 (0.34)	2.32 (2,16)	0.13	0.65	67
Power P3	−1.96 (0.27)	−1.84 (0.33)	−1.92 (0.34)	2.29 (2,16)	0.13	0.43	78
Power P4	−1.95 (0.39)	−1.82 (0.45)	−1.87 (0.33)	0.66 (2,16)	0.53	0.31	78

Cohens d calculated between T0 and T1. Positive Cohen’s d mean improved function;  %improvement:  % of athletes who increased between T0 and T1

### Follow-Up

At the 5-week follow-up (T2), no significant declines in subjective and objective outcome parameters were detected compared to T1. Compared to group A, non-significant decreases were however detected in self-reported performance (7.2–6.3) and sleep quality (3.6–4.1). SDNN, RMSSD and LF and HF power decreased non-significantly to values approximating the baseline value. Alpha power in general was maintained at T2, compared to T1.

## Discussion

The main objective of this study was to explore if HRV-feedback or neurofeedback combined with a peak performance program would lead to a change in HRV and EEG outcomes and consequently, in self-reported factors related to stress, recovery and sleep quality in elite sportsmen and women. The current results point toward the notion that a combination of a group based peak performance program with either HRV-feedback or neurofeedback may lead to changes in performance-related outcomes and stress reduction. For Group A the EEG alpha power and LF/HF ratio improved and SIM60 emotional stability and concentration indices revealed better scores after intervention. For the athletes HRV low frequency power and recovery index of the RESTQ significantly improved. Overall, the largest changes appeared to be present in the HRV and EEG measures, and little significant changes were identified on clinically relevant self-report measures. This may imply that changes in HRV and EEG parameters not automatically imply better scores on performance indices. However, all non-significant differences showed a tendency towards improvement.

The literature on mental stress and peak performance is extensive. Both peak performance or stress reduction programs are known to increase relevant outcomes on HRV and performance measures (Hammermeister and VonGuenthner 2005; Jones and Hardy 1989). For biofeedback, and especially HRV and alpha power feedback, the literature is contradictory and frequently of sub-optimal quality. Fluctuating lower and upper theta power values appeared predictive for unsuccessful throws in basketball players, suggesting that a stable attention and arousal may facilitate athletic performance (Chuang et al. 2013). In one high quality study that investigated the effects of alpha power training in gymnasts, small but significant changes were identified in sleep quality, mental and physical shape (Dekker et al. 2014). The authors stretch the importance of appreciating relatively small and non-significant changes, because a small change may lead to the difference between winning and losing in mentally demanding sports. Which effect sizes are relevant for athletes is unknown, because there are no standard cut-off points for clinical relevance. While non-significant, a change for 6.4–7.2 on the NRS for self-reported performance may be relevant for athletes. The p-value in this case is not the best way of interpreting the results. An alternative has previously been suggested by Paton and Hopkins 2005, who reports that a change in performance outcome of 1 % is frequently described as the smallest worthwhile change (SWC) (Paton and Hopkins 2005). Calculation of the SWC however, appeared not feasible for this study. A change of 1 % in a NRS 0–10 scale is not feasible to measure in many intermediate outcome measures and falls within minimal detectable change of the NRS, which is about 2.0 points (Ostelo et al. 2008). SWC values of the used outcome measures are currently unknown. Therefore, we chose to present a Cohen’s d as effect size (Cohen 1988) and we reported how many players improved per outcome. This may provide trainers an indication whether or not this training is worth the efforts.

The fact that both groups revealed improvements on HRV measures and Group A also on EEG alpha power supports the idea that there is a large common ground in which both feedback training interventions work. This is according to previous research, that reported on the effects of alpha power EEG training on an increase in HRV, depending on the baseline alpha power of subjects (Bazanova et al. 2013), while on the other hand, training on respiratory sinus arrhythmia leads to significant changes in overall alpha power (Sherlin et al. 2010). This may imply that training the brain or the heart, may lead to changes in both. For the current study, however, we cannot rule out that the improvements are only caused by the coaching program. Randomized trials should be performed to identify which of the program ingredients have the most effect.

### Strengths

To the authors knowledge, this is the first study to investigate a combination of two frequently used interventions to increase performance and reduce mental stress. The fact that the current effects are larger compared to previous effects found for single interventions, supports the hypothesis that a combination of peak performance training and biofeedback reinforces the positive effects of either intervention. The aim of this study was to gain a high external validity and provide a training procedure of two best practices combined. The strength of this study were the standardized measures of ECG and EEG and validated self-reported questionnaires in this study, which limited the measurement error. In the current study we show that a combination of different approaches to influence stress reduction and mental capacity during sports have a positive effect.

### Limitations

There are several limitations of this study. The quality of evidence is rated as level C. Two groups were tested and could not be compared to each other because of differences in characteristics (gender, age and sportstype) and intervention (neurofeedback and heart rate feedback). Because no control group existed, causality could not be determined. The number of included participants was limited (N = 11 and N = 10). While trends in improvement on basically all outcomes were observed, significance may not have been reached because of the low number of cases included. It remains unclear whether effects were generated because of placebo, coaching, training effects, or specific biofeedback training.

This study was regarded a pilot study because of the above mentioned limitations in design. A sample size calculation on the recovery scale of the RESTQ reveals that 42 subjects are needed per arm to find significant differences of 7.0 points (α = 0.05) with a beta of 0.1. Future trials will need these many subjects.

It can be concluded that a mental coaching program combined with either HRV or EEG alpha power feedback may increase HRV and alpha power and may lead to better performance-related outcomes and stress reduction. Further research is needed to elucidate the effects of either type of feedback, to compare effects with a control group and reduce confounding.
